# Promising Role of Growth Hormone-Boosting Peptide in Regulating the Expression of Muscle-Specific Genes and Related MicroRNAs in Broiler Chickens

**DOI:** 10.3390/ani11071906

**Published:** 2021-06-26

**Authors:** Doaa Ibrahim, Hanan S. Al-Khalaifah, Ahmed Abdelfattah-Hassan, Haitham Eldoumani, Safaa I. Khater, Ahmed H. Arisha, Sally A. M. Mohamed, Tamer Ahmed Ismail, Samar A. Tolba

**Affiliations:** 1 Department of Nutrition and Clinical Nutrition, Faculty of Veterinary Medicine, Zagazig University, Zagazig 44511, Egypt; samartolba5@gmail.com; 2 Environment and Life Sciences Research Center, Kuwait Institute for Scientific Research, P.O. Box 24885, Safat 13109, Kuwait; hkhalifa@safat.kisr.edu.kw; 3 Department of Anatomy and Embryology, Faculty of Veterinary Medicine, Zagazig University, Zagazig 44511, Egypt; aabdelfattah@vet.zu.edu.eg; 4 Biomedical Sciences Program, University of Science and Technology, Zewail City of Science and Technology, October Gardens, 6th of October, Giza 12578, Egypt; 5 Department of Anatomy and Embryology, Faculty of Veterinary Medicine, Mansoura University, Mansoura 35516, Egypt; hdomany@mans.edu.eg; 6 Department of Biochemistry, Faculty of Veterinary Medicine, Zagazig University, Zagazig 44511, Egypt; safaa_khater83@yahoo.com; 7 Department of Physiology, Faculty of Veterinary Medicine, Zagazig University, Zagazig 44511, Egypt; vetahmedhamed@gmail.com; 8 Department of Animal Physiology and Biochemistry, Faculty of Veterinary Medicine, Badr University in Cairo (BUC), Badr City 11829, Egypt; 9 Department of Histology and Cytology, Faculty of Veterinary Medicine, Zagazig University, Zagazig 44511, Egypt; sally_shehata2011@yahoo.com; 10 Department of Clinical Laboratory Sciences, Turabah University College, Taif University, P.O. Box 11099, Taif 21944, Saudi Arabia; t.ismail@tu.edu.sa

**Keywords:** muscle development, myomiR, gene expression, synthetic peptide, broiler

## Abstract

**Simple Summary:**

In chickens, breast muscle is a key contributor to meat yield. Skeletal muscle development is a complex process regulated by many genes, transcription factors, and miRNA through different muscle signaling pathways. The latter are activated by synthetic growth hormone-boosting peptides (GHBP) that mimic the effects of endogenous growth factors, mainly insulin-like growth factor-1 (IGF-1)/mammalian target of rapamycin (mTOR), and modulate myostatin gene expression. The molecular mechanisms underlying chicken breast muscle development in relation to GHBP are still unclear. In the current study, the role of GHBP in a chicken’s growth rate, skeletal muscle development-related genes and myomiRs, serum bio-chemical parameters, and myofiber characteristics post-hatching was evaluated. Using of GHBP at a level of 200 μg/kg positively regulated gene expression related to muscle growth and muscle-specific myomiRs. This was evidenced by upregulation of IGF-1, mTOR, myoD and myogenin genes, and miR-27b and miR-499 and downregulation of myostatin, Pax-3 and -7 genes and miR-1a, miR-133a, miR-133b, and miR-206 compared to the control group. Therefore, administration of GHBP at the level of 200 μg/kg to broiler chicks can accelerate their growth by enhancing skeletal muscle development via controlling the transcription of breast muscle-related genes and associated myomiRs.

**Abstract:**

Appropriate skeletal muscle development in poultry is positively related to increasing its meat production. Synthetic peptides with growth hormone-boosting properties can intensify the effects of endogenous growth hormones. However, their effects on the mRNA and miRNA expression profiles that control muscle development post-hatching in broiler chicks is unclear. Thus, we evaluated the possible effects of synthetic growth hormone-boosting peptide (GHBP) inclusion on a chicken’s growth rate, skeletal muscle development-related genes and myomiRs, serum biochemical parameters, and myofiber characteristics. A total of 400 one-day-old broiler chicks were divided into four groups supplied with GHBP at the levels of 0, 100, 200 and 300 μg/kg for 7 days post-hatching. The results showed that the highest levels of serum IGF-1 and GH at d 20 and d 38 post-hatching were found in the 200 μg/kg GHBP group. Targeted gene expression analysis in skeletal muscle revealed that the GHBP effect was more prominent at d 20 post-hatching. The maximum muscle development in the 200 μg/kg GHBP group was fostered by the upregulation of IGF-1, mTOR, myoD, and myogenin and the downregulation of myostatin and the Pax-3 and -7 genes compared to the control group. In parallel, muscle-specific myomiR analysis described upregulation of miR-27b and miR-499 and down-regulation of miR-1a, miR-133a, miR-133b, and miR-206 in both the 200 and 300 μg/kg GHBP groups. This was reflected in the weight gain of birds, which was increased by 17.3 and 11.2% in the 200 and 300 μg/kg GHBP groups, respectively, when compared with the control group. Moreover, the maximum improvement in the feed conversion ratio was achieved in the 200 μg/kg GHBP group. The myogenic effects of GHBP were also confirmed via studying myofiber characteristics, wherein the largest myofiber sizes and areas were achieved in the 200 μg/kg GHBP group. Overall, our findings indicated that administration of 200 μg/kg GHBP for broiler chicks could accelerate their muscle development by positively regulating muscle-specific mRNA and myomiR expression and reinforcing myofiber growth.

## 1. Introduction

In chickens, skeletal muscles constitute about 50% of total body weight, with a significant influence on edible meat yield and livestock production [1]. Skeletal muscle development is a multi-step process involving the formation and hypertrophy of myofibers during embryogenesis and post-hatching [2]. Earlier literature interested in the mechanisms involved in muscle development has highlighted two important signaling pathways: insulin-like growth factor-1 (IGF-1)/mammalian target of rapamycin (mTOR) and the myostatin-signaling pathway [3]. The balance between these two pathways has been found to be crucial for maintaining normal skeletal muscle formation and development [4]. The IGF-1/mTOR signaling pathway is a positive regulator of skeletal muscle growth and is associated with small, non-coding RNAs (miRNAs or miRs) such as miR-1a and -133a [5]. In the same context, the myostatin-signaling pathway is a negative regulator of skeletal muscle growth and has been found to be associated with miR-27b and miR-499 [3,6]. Muscle-associated miRNAs mediate gene silencing and play vital roles to fine-tune the expression of genes controlling myogenesis; they act via their multi-interactions with muscle mRNAs within muscle intricate-signaling pathways [7].

Previous literature has reported that animal growth is mainly coordinated by somatotropic-axis hormones including growth hormone-releasing hormone (GHRH), IGFs, growth hormone (GH), and somatostatin [8]. These hormones regulate skeletal muscle growth via modulating the major anabolic endocrine pathway, i.e., the IGF-1/mTOR pathway [8]. Very recently, the potential roles of bioactive peptides mimicking GH properties for boosting the efficiency of animal and aquaculture production became a hotspot for research [9,10]. Because of their growth-promoting properties, bioactive peptides are favorably being used in animal feed. Furthermore, in the chicken production industry, using more safe dietary alternatives to antibiotic growth boosters is becoming particularly crucial [11,12]. Nevertheless, the impact of such peptides on poultry productivity is still not fully investigated, and more information is still necessary. Synthetic GH secretagogues (GHSs) or GH-releasing peptides (GHRP) comprise synthetic peptides that have potent GH-boosting properties and effectively mimic the influence of endogenous GH and, in turn, stimulate animal growth [9,13]. In various mammalian species, the administration of GHSs has been shown to promote feed intake (FI) [9,13,14,15,16]. However, earlier research in neonatal chicks showed that GHRP-6 injection suppressed FI but not feed retention [17]. Therefore, these intriguing reports drive the need for deeper understanding of the mechanism(s) underlying GHSs’ and GHRPs’ effects in chickens. Although a few studies showed that GHRPs could effectively induce GH and IGF release and boost animal growth [14,18], to our best knowledge, no studies have systematically investigated the impact of different dietary GHRP levels on muscle growth, muscle-related genes, and muscle-specific miRNAs (myomiRs) expression in chickens.

Therefore, the current study aimed to explore the importance of administering GHBP to broiler chicks during the first 7 days post-hatching on chicks’ skeletal muscle growth and overall growth performance throughout the total growing period. In addition, we aimed to highlight the different molecular mechanisms elicited by GHBP through exploring the expression of different myogenesis-related mRNAs and myomiRs.

## 2. Materials and Methods

### 2.1. Birds, Diets, and Management

The current study was conducted at the poultry research farm of the Department of Nutrition and Clinical Nutrition, Faculty of Veterinary Medicine, Zagazig University, with 400 one-day-old, unsexed Ross-308 broiler chicks. The animal protocol was accepted by the Institutional Animal Care and Use Committee of Zagazig University, Egypt (Approval No. ZU-IACUC/F/116/2020). The birds were acquired from a commercial hatchery and were assigned in a completely randomized design to four experimental treatments (10 replicates per experimental treatment and 10 birds per treatment replicate). The birds were fed a corn–soybean meal basal diet un-supplied (control group) or supplied with GHBP (Hexarelin, product No; 80666-50MG, Sigma Aldrich) at the level of 100, 200 or 300 μg/kg of diet during the first 7 days of the chick’s life. Starting from day 8, all birds in all treatments were fed the same diet. The concentrations of GHBP in diets before feeding were checked by high-performance liquid chromatography (HPLC). The peptides were encapsulated with chitosan and alginic acid, according to the protocol of [10], dissolved in 5 mL saline, and sprayed over the diet at the time of feeding. The starter diet was offered to the birds until 10 days of age and then the grower and finisher diets from days 11 to 22 and 23 to 38, respectively, following the standard procedures of Ross 308 broiler nutrition specifications [19] (Table 1).

Feed and fresh water were given ad libitum to all birds throughout the study (38 days). The birds were kept under the same management and environmental conditions. The lighting system for the first 3 days of the study was set as 23:1 h light:dark and then changed to 16:8 h light:dark until the end of the experiment. 

### 2.2. Growth Performance

The average body weight was recorded upon birds’ arrival and on day 10, 21, and 38 of the experiment to determine average body weight (BW); next, body weight gain (BWG) was calculated. Also, the difference between both the weight of the provided feed and the remaining residues, feed intake (FI), was determined per replicate. The feed conversion ratio (FCR) was then calculated as we previously reported [20,21,22]

### 2.3. Sample Collection

At day 20, birds from each group were randomly chosen for blood and tissue sampling (*n* = 10/group). Blood was collected from the wing vein into 3 mL vacutainer tubes (BD Vacutainer, Becton Dickinson, Franklin Lakes, NJ, USA). The serum was obtained by centrifuging samples for 15 min at 3000× *g* and then kept at −20 °C for GH and IGF-1 analyses. The same birds were slaughtered according to the recommendations of the institutional committee, and a part of the breast muscle (Pectorales major) was collected, immediately frozen in liquid nitrogen, and then stored at −80 °C for mRNA and miRNA expression assay.

At the end of the experiment (day 38), the same procedures of day 20 were followed for collecting blood and tissue samples (*n* = 10/group), in addition to histological samples from the breast muscle, for further biochemical and molecular analysis. 

### 2.4. Serum Biochemical Analysis

Serum total cholesterol (TC), triglycerides (TGs), high-density lipoprotein cholesterol (HDL-C), low-density lipoprotein cholesterol (LDL-C), very-low-density lipoprotein cholesterol (VLDL-C), creatinine, uric acid, aspartate aminotransferase (AST), and alanine aminotransferase (ALT) were determined using commercial diagnostic kits (Sigma-Aldrich, Inc., St. Louis, MO, USA). Serum GH and insulin-like growth factor-1 (IGF-1) were assessed using radioimmunoassay kits (Nanjing Jiancheng Technology Co., Ltd., Nanjing, China), according to ROH, et al. [23].

### 2.5. Total RNA Isolation and MRNA Expression of Marker Genes

Using TRIzol reagent (Life Technologies, Carlsbad, CA, USA), total RNA was extracted and purified from muscle tissue (approx. 30 mg), and then extracted RNA was treated with DNase I (DNase I recombinant, RNase-free, cat. no. 04716728001, Roche Diagnostics GmbH, Mannheim, Germany) and purified a second time with TRIzol reagent. RNA quantity and quality were analyzed using NanoDrop^®^ ND–1000 spectrophotometer (NanoDrop Technologies; Wilmington, DE, USA). Complementary DNA (cDNA) was obtained by reverse transcription of isolated RNA samples using RevertAidTM H Minus kits (Fermentas Life Science, Pittsburgh, PA, USA). The cycling condition for cDNA amplification was at 37 °C for 60 min activation followed by heating to 95 °C for 5 min, and finally, a holding temperature of 4 °C (Averiti 96-well thermal cycler, Applied Biosystems, Foster City, CA, USA). The abundance of mRNA of the key genes involved in muscle development and protein production including myostatin (MSTN), myogenin (MyoG), myogenic determination factor1 (MyoD1), paired-box3 (Pax3), Pax7, IGF-1, and the mammalian target of rapamycin (mTOR) were determined by qPCR using the PrimeScript™ RT Reagent kit (Takara Bio Inc., Shiga, Japan) and SYBR Green qPCR Master Mix (Applied Biosystems, Foster City, CA, USA). Briefly, the reaction mixture consisted of 4 µL sample cDNA, 12.5 μL SYBR Green master mix, 1.5 µL of forward primer (10  pmol), 1.5 µL reverse primer (10  pmol), and 5.5 µL of nuclease-free water. The optimized thermal cycling conditions were as follows: initial denaturation at 95 °C for 15 min/one cycle, 95 °C for 15 s for denaturation, 60 °C for 60 s for annealing, and extension at 72 °C for 60 s for 40 cycles, with data acquisition occurring at the 60 °C step. Primer sequences for the assessed genes are listed in Table 2. The glyceraldehyde-3-phosphatedehydrogenase (GAPDH) gene was used as an internal control gene for data normalization. The results of relative fold changes in the expression of target genes were estimated by using the 2^−ΔΔCt^ method [24].

### 2.6. Evaluation of MiRNA Expression

The cDNA was performed following the TaqMan™ microRNA assay protocol (ThermoFisher Scientific, Waltham, MA, USA) in a Veriti 96-well thermal cycler (Applied Biosystems, Foster City, CA), following manufacturer instructions. The Stem-loop RT and miRNA specific primers and the universal reverse primer were designed using assay design software (http://genomics.dote.hu:8080/mirnadesigntool, accessed on 5 October 2019) [25]. All primers were synthesized by Sangon Biotech (Beijing, China). Next, miRNA expression of key regulators involved in muscle mass growth and development including miR-1a, -27b, -133a, -133b, -206, and -499 were identified by quantitative, real-time PCR (qPCR) using a reaction master mix of 12.5 μL Maxima SYBR green/ROX qPCR Master Mix (2X), 1 μL forward primer, 1 µL Stem-loop RT primer, 1 μL universal reverse primer, 1 μL cDNA, and 8.5 μL nuclease-free water in a Mx3005P Real-Time PCR System (Agilent Stratagene, Santa Clara, CA, USA), using Maxima SYBR Green/ROX qPCR Master Mix (2×) (ThermoFisher Scientific, Waltham, MA, USA), following the manufacturer’s instructions. All RT-qPCR processes and reporting complied with MIQE guidelines [26]. The relative expression of each gene was normalized to chicken U6 and reported as fold change relative to the control using the 2^−ΔΔCT^ method [24]. Table 3 shows all sequences for the used primers in evaluating miRNA expression.

### 2.7. Muscle Fiber Histological Characteristics

Skeletal muscle samples were obtained from the breast muscle (Pectorales major) of 10 random chickens per group (the same broilers used for sampling at day 38), washed in warm PBS, and immediately fixed in neutral buffered formalin (10%) solution for at least 24 h. Next, the specimens were dehydrated in alcohol, cleared in xylene, and embedded in paraffin, and the blocks were cut into 5 μm sections. The sections were stained using standard Hematoxylin and Eosin (H&E) stain, mounted with coverslips, and viewed under light microscopy [27]. Five photographs from each slide were taken for muscle cross-sections to allow measuring the cross-sectional area (µm^2^) of myofibers and counting the total number of fibers (TNF) per field. The myofiber cross-sectional area and the number of myofibers in each image was calculated using ImageJ software (National Institute of Health, Bethesda, MD, USA).

### 2.8. Statistical Analysis

All data were analyzed by one-way analysis of variance using SPSS version 20.0 for Windows (SPSS, Inc., Chicago, IL, USA). The replicate was considered as an experimental unit (*n* = 10). Using Tukey’s honest significant difference test, the significant differences between mean values were analyzed. Pooled SEs were identified for all the analyses, and the level of significance was set at *p* < 0.05.

## 3. Results 

### 3.1. Growth Performance 

The effects of dietary GHBP on broilers’ growth performance are listed in Table 4. BW and BWG were increased (*p* < 0.05) in all GHBP-supplied groups during the starter period; however, compared to all treated groups, the group supplied with GHBP at 200 and 300 μg/kg decreased FI (*p* < 0.05). FCR was improved (*p* < 0.05) in all GHBP-supplied groups, with the highest significant level detected in the group supplied with 200 μg/kg. During the grower period, BW was elevated (*p* < 0.05) and FCR was decreased (*p* < 0.05) in all GHBP-supplied groups, with the highest significant level reported at the level of 200 μg/kg; however, the FI showed an increase (*p* < 0.05) at the level of 200 μg/kg in comparison to other experimental groups. The BWG was enhanced (*p* < 0.05) in the group supplied with 200 and 300 μg GHBP/kg. During the finisher and the overall performance periods, BW and BWG were elevated (*p* < 0.05) and FCR was decreased (*p* < 0.05) in all GHBP supplied groups, with the highest significant level reported at the level of 200 μg/kg. The FI did not show a significant change during the finisher and for the overall period.

### 3.2. Blood Biochemical Parameters

Data regarding the impact of GHBP on blood biochemical parameters are shown in Table 5. The GHBP-supplied groups did not show a significant change in serum AST, ALT, creatinine, uric acid, TGs, or VLDL-C concentrations. However, in all the supplied groups, the TC and LDL-C concentrations decreased (*p* < 0.05) with an increase in HDL-C level compared with the control group. The serum level of IGF-1 was elevated (*p* < 0.05) in all GHBP-supplied groups, with the most pronounced effect obtained at 200 μg GHBP/kg at both 20 and 38 days. The groups supplied with 200 or 300 μg GHBP/kg showed an increase (*p* < 0.05) in GH level at 20 days; however, such an increase (*p* < 0.05) was detected in all GHBP-supplied groups in comparison with the control group.

### 3.3. Muscle Development-Related Gene Expression

**Figure 1:**
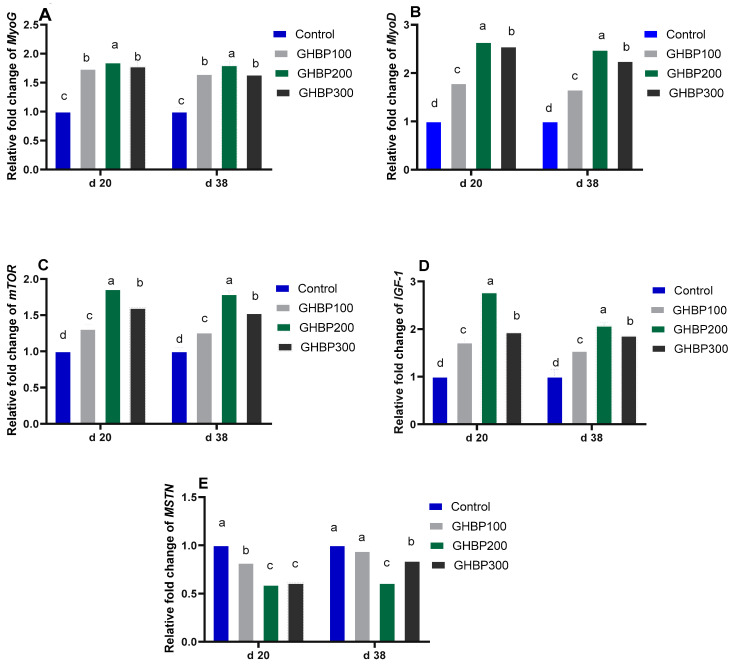
Real-time qPCR analysis for muscle development-related genes (*MyoG (***A**); *MyoD* (**B**); *MTOR* (**C**); *IGF-1* (**D**); *MSTN* (**E**)) expression in broiler chickens supplied with GHBP at day 20 and day 38 of age. GHBP: growth hormone-boosting peptide; MyoD: myogenic determination factor; MyoG: myogenin; MSTN: myostatin; mTOR: mechanistic target of rapamycin; IGF-1: insulin-like growth factor 1. Bars with different letters above them correspond to values that were significantly different (*p* < 0.05). Control: basal diet without GHBP; GHBP100: basal diet plus GHBP at a level of 100 μg/kg; GHBP200: basal diet plus GHBP at a level of 200 μg/kg; GHBP300: basal diet plus GHBP at a level of 300 μg /kg. ^a,b,c,d^ Means with different superscripts within the same row differ significantly (*p* < 0.05).

**Figure 2:**
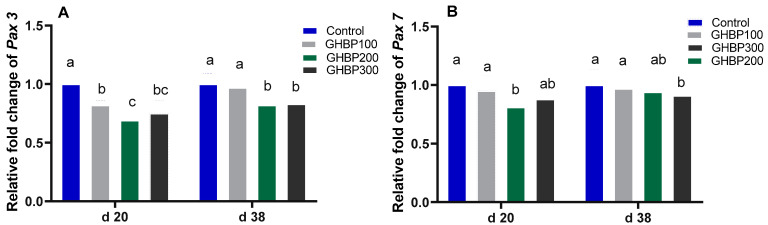
Real-time qPCR analysis for muscle development-related gene (*Pax 3 (***A**), and *Pax 7* (**B**)) expression in broiler chickens supplied with GHBP at d 20 and d 38 of age. GHBP: growth hormone-boosting peptide; Pax: paired-box. Bars with different letters above them correspond to values that were significantly different (*p* < 0.05). Control: basal diet without GHBP; GHBP100: basal diet plus GHBP at a level of 100 μg/kg; GHBP200: basal diet plus GHBP at a level of 200 μg/kg; GHBP300: basal diet plus GHBP at a level of 300 μg /kg. ^a,b,c,^ Means with different superscripts within the same row differ significantly (*p* < 0.05).

Real-time qPCR analysis for muscle development-related genes is shown in Figure 1 and Figure 2. Compared with the control group, the GHBP-supplied groups showed an increase (*p* < 0.05) in mRNA expression levels of MyoG, IGF-1, mTOR, and MyoD, with the highest significant level obtained at 200 μg GHBP/kg at both 20 and 38 days. In addition, compared with the control group, MSTN and Pax3 mRNA abundances were decreased (*p* < 0.05) in all GHBP-supplied groups at 20 days; however, at 38 days, such a decrease (*p* < 0.05) was detected in the groups supplemented with 200 or 300 μg GHBP/kg. The 200 or 300 μg GHBP/kg lowered (*p* < 0.05) the mRNA expression level of Pax7 at 20 and 38 days. 

### 3.4. Integrated Analysis of MiRNAs Related to Muscle Mass Development 

**Figure 3:**
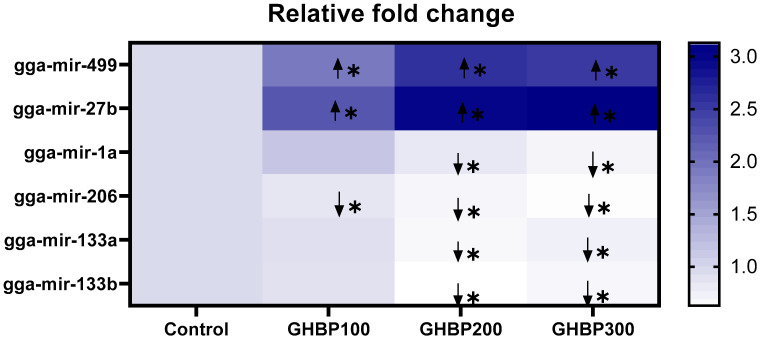
Heat map for expression of myomiRs (miRs-1a, 133a, 133b, 27b, and 499) in broiler chickens supplied with GHBP at day 38 of age. GHBP: growth hormone-boosting peptide. Data are expressed as means ± SE. Bars with different letters above them correspond to values that were significantly different (*p* < 0.05). Control: basal diet without GHBP; GHBP100: basal diet plus GHBP at a level of 100 μg/kg; GHBP200: basal diet plus GHBP at a level of 200 μg/kg; GHBP300: basal diet plus GHBP at a level of 300 μg /kg. * Squares correspond to values that are significantly different. Other squares with no (*) denote no significant changes in of miRNA expression among the experimental groups. 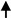 Correspond to upregulation of miRNA expression relative to the control group (*p* < 0.05). 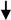 Correspond to downregulation of miRNA expression relative to control group (*p* < 0.05).

In comparison with the control, all GHBP-supplied groups showed an increase (*p* < 0.05) in miR-27b and miR-499 expression levels; however, they showed a decrease (*p* < 0.05) in their miR-206 expression level. The groups supplied with 200 or 300 μg GHBP/kg showed a decrease (*p* < 0.05) in the expression levels of miR-1a, miR-133a, and miR-133b (Figure 3).

### 3.5. Muscle Fiber’s Characteristics

**Figure 4:**
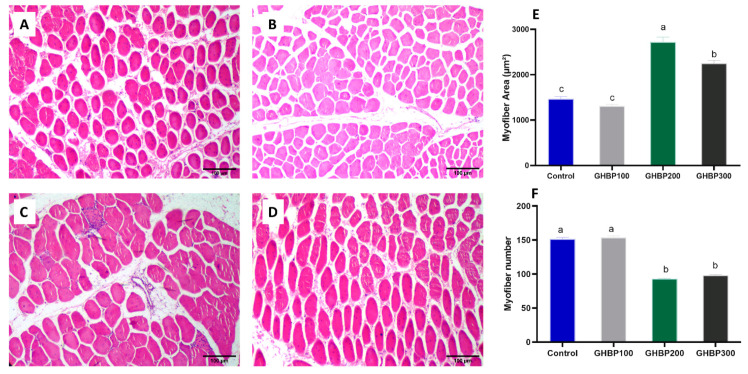
Breast muscle histological cross sections of broiler chickens supplied with GHBP: control (**A**), GHBP100 (**B**), GHBP200 (**C**), and GHBP300 (**D**). Muscle fiber’s characteristics: myofiber area (µm^2^) (**E**) and myofiber number per field (**F**). GHBP: growth hormone-boosting peptide. Data are expressed as means ± SE. Bars with different letters above them correspond to values that were significantly different (*p* < 0.05). Control: basal diet without GHBP; GHBP100: basal diet plus GHBP at a level of 100 μg/kg; GHBP200: basal diet plus GHBP at a level of 200 μg/kg; GHBP300: basal diet plus GHBP at a level of 300 μg /kg. ^a,b,c,^ Means with different superscripts within the same row differ significantly (*p* < 0.05).

The myofiber histological characteristics (area in µm^2^ and myofibers number) of the studied groups are shown in Figure 4. The myofibers of the group supplied with 200 μg/kg GHBP showed the highest myofiber’s area (average area = 2720 µm^2^, *p* < 0.01, Figure 4E), and the lowest total number of myofibers per field (average number per field = 93 myofibers, *p* < 0.01, Figure 4F). While the mean area of myofibers in the groups supplied with 300 μg/kg GHBP was significantly lower (2246 µm^2^, *p* < 0.01) than 200 μg/kg GHBP group, it was significantly higher (*p* < 0.01) than both 100 μg/kg GHBP and control groups. There was no statistical difference in the average myofiber area of the 100 μg/kg GHBP and control groups (1306 and 1463 µm^2^, respectively, *p* > 0.05). The total number of myofibers per filed was significantly lower (*p* < 0.01) in the 300 and 200 μg/kg GHBP groups (98 and 93 TNF/field, respectively) compared to the 100 μg/kg GHBP and control groups (153 and 151 TNF/field, respectively).

## 4. Discussion

Skeletal muscle development in poultry is closely related to the GH–IGF system as a crucial promoter of growth. Post-hatching, the potential role of synthetic peptide-boosting growth hormone on muscle development in broiler chicks is still limited. Therefore, it is important to determine different molecular mechanisms (at the mRNA and miRNA levels) that control the post-hatching development of chicken skeletal muscle following GHBP supplementation.

Our findings revealed an enhanced growth performance in terms of improved BW, BWG, and FCR in response to GHBP supplementation during the first week of broilers’ lives, particularly when using GHBP at the level of 200 μg/kg. To the best of our knowledge, the obtained results in this study are the first to be recorded in broilers in response to GHBP administration. However, other research groups have reported similar effects of dietary GHBP in other species, who attributed the effect of GHBP on animals’ growth performance to the somatotropic axis hormones, GH/IGF-1 [9,15,16]. Notably, the maximum growth-promoting effect of GHBP was observed in the group supplemented with 200 μg/kg; however, with increasing the levels of GHBP to 300 μg/kg, the growth rate was decreased. The lack of growth stimulation in the 300 μg/kg-supplemented group may reflect decreasing GH response to the higher administration of GHBP [28]. Additionally, administration of higher doses of GHBP were responsible for negative feedback on GHRP-mediated receptors, causing a decrease in the stimulation of growth hormone secretion [29]. 

Thus far, our GH and IGF-1 hormonal level findings may indicate a possible positive relationship between GHBP supplementation, serum GH/IGF-1, and chickens’ growth performance. However, the broilers’ metabolic response in our study did not show substantial effects except for the TC, LDL-C, and HDL-C serum levels. It appeared that dietary GHBP mainly altered lipid profile indices; nevertheless, no shift in VLDL-C and TGs implied a lack of consistent impact on the serum lipid profile. This suggests that evaluating GHBP’s effect on blood lipid profiles by testing these changes only in serum without assessing internal organs might not be a reliable tool for determining the true metabolic impact of dietary GHBP in broilers and requires further investigation.

Because skeletal muscles in chickens constitute about 50% of total BW, with a significant influence on edible meat yield and livestock production [1], we investigated the genes expression and miRNA levels of vital regulators of myogenesis as well as protein production and deposition in response to GHBP supplementation to understand such mechanism(s) in broilers. In effect, GHBP caused an increase in mRNA expression levels of *MyoG*, *IGF*-1, *mTOR*, and *MyoD1* and the expression of miR-27b and miR-499. In contrary, it caused a reduction in *MSTN*, *Pax3*, and *Pax7* mRNA abundances and miR-1a, -133a, -133b, and -206 expression levels. As far as we know, none of the previous research that supplemented GHBP in different species’ diets investigated its impact in relation to muscle development and protein synthesis; it has only focused on GHBP’s effect on animals’ feeding behavior, growth performance, and somatotropic axis hormone secretions [9,17,30,31]. Thus, the current findings are the first reports to characterize supplemental GHBP’s impact on the molecular response of avian muscle development and protein production. GHBP supplementation resulted in upregulation of the mRNA level of most of the determined genes known to play vital roles in controlling muscle growth and development. Such muscle growth mainly relies on accumulation of protein in myofibers, which is induced by activation of the mTOR signaling pathway. mTOR activation is controlled by different regulators such as IGF-1, nutritional status, and energy balance [30]. In this study, GHBP supplementation significantly upregulated *IGF*-1 mRNA expression, which activated the IGF-1 receptor located in the skeletal muscles, which, in turn caused an activation of downstream factor *mTOR* to promote protein accumulation [31]. 

The key muscle-specific miRNAs, miR-1a, -133a, -133b, and -206, which formed a regulatory feedback loop with the assessed protein production regulator genes, showed decreased expression in the current study. These findings agree with McCarthy and Esser [5], who stated that miR-1a and -133a expression levels were decreased during skeletal muscle hypertrophy, which contributed to the *IGF*-1/*mTOR* activation pathway. However, Ge and Chen [32] reported that miR-1a and -206 expression levels were boosted during myogenesis to promote the differentiation of satellite cells and suppress their proliferative potential through targeting the *Pax7* gene, which played a leading role in satellite cell proliferation and initiation of the myogenesis program [33]. In spite of the vital role of *Pax7* and its close homologue, *Pax3*, in working upstream of *MyoD* to trigger the expression of the skeletal muscle gene and induce myogenesis, earlier gene targeting studies showed that persistent *Pax7* expression in satellite cells delayed myogenesis, and elevated *Pax7* expression in primary myoblasts restricted *MyoD* expression, which prevented *myogenin* induction and terminal skeletal muscle differentiation [34]. To date, the decreased *Pax7* and 3 and elevated *MyoG* and *MyoD1* mRNA expression levels in our study are consistent with the mechanisms reported from earlier research [33,34] and suggest that GHBP supplementation has positive effects on the post-hatch growth of broilers’ skeletal muscle. 

On the other hand, we reported significant changes in the expression of the key regulators controlling the myostatin-signaling pathway. Myostatin, a negative regulator of skeletal muscle growth, showed decreased mRNA expression level in our study. We also reported an increased expression level of the miRNAs associated with the myostatin-signaling pathway, miR-27b and miR-499. Indeed, myostatin is known to inhibit the proliferation and differentiation of myoblasts, which, in turn, maintain the quiescent state of satellite cells post-hatch [35]. The upregulated miR-27b have been reported to reduce the stability of *MSTN* mRNA post-transcriptionally, which consequently enhances differentiation of satellite cells and skeletal muscle mass formation [6]. Interestingly, Crist et al. [36] stated that another mechanism of enhancement of satellite cell differentiation by miR-27b was through the downregulation of the *Pax3* mRNA level, which agrees with our findings. Likewise, increased miR-499 expression has been reported to be associated with myostatin expression in human skeletal muscles [37]; however, further studies are still required to investigate the relationship between miR-499 and *MSTN* mRNA in regulating avian skeletal muscle mass.

The increase in meat production in broiler chicks depends mainly on the growth of their skeletal muscles [38]. Post-hatching, skeletal muscles in broiler chicks grow mainly by increasing the size, diameter and length of their myofibers [39]. Previous studies have established a positive correlation between myofiber characteristics and muscle growth of broiler chicks [40,41,42]. In these studies, concerning myofiber characteristics, higher body weight in fast-growing chicks [42] or higher breast muscle weight [41] was mainly associated with larger myofiber diameter and area. Therefore, larger body weight in chicken is achieved by increasing the myofibers’ area, which, consequently, results in less density of fibers per area of a given muscle [43]. In accordance, the group supplied with 200 μg/kg GHBP in our study attained higher body weight and showed better muscle characteristics with the highest area and, therefore, the least number of myofibers per field in all the studied groups. Compared to the 200 μg/kg group, the effect of GHBP did not improve when using higher doses (i.e., 300 μg/kg), while the addition of 100 μg/kg showed less improvement in myofiber characteristics and body weight. Similarly, accelerated growth and similar improvement in myofiber observations were achieved in yaks supplied with growth hormone-releasing peptide-2, which enhanced muscle protein deposition and upregulated protein synthesis pathways in myofibers [9].

In conclusion, our study showed that dietary GHBP exerted a dose-dependent but nonlinear impact on the physiological and molecular profiles of key regulators that controlled broilers’ skeletal myogenesis and muscle mass growth. The supplemental GHBP in the pre-starter diets for broilers improved the chickens’ growth performance; somatotropic axis hormones, GH/IGF-1; and increased the expression of regulators that controlled skeletal muscle development, protein production, and deposition (*MyoG*, *MyoD*, *IGF*-1, and *mTOR*) through the decreased expression of miR-1a, -133a, -133b, and -206. In contrast, a decrease in myostatin gene expression was detected, which proposed to be through the upregulation of miR-27b and -499. 

## Figures and Tables

**Table 1:** Ingredients and composition (%) of the basal diet.

Ingredients	Starter (0–10 days)	Grower (11–22 days)	Finisher (23–38 days)
Yellow corn	53.03	56.6	59.90
Soybean meal, 44%	33.10	29.1	25.12
Corn gluten, 60%	7.04	7.05	7.00
Soybean oil	2.40	3.20	4.11
Limestone	1.52	1.36	1.28
Monocalcium phosphate	1.80	1.65	1.53
Common salt	0.38	0.38	0.38
Premix *	0.30	0.30	0.30
DL-methionine, 98%	0.13	0.11	0.11
Lysine, Hcl, 78%	0.25	0.21	0.22
Antitoxin	0.05	0.05	0.05
Chemical composition			
ME, Kcal/Kg	3000.95	3100.02	3200.40
CP%	23.01	21.50	20.00
EE%	4.86	5.76	6.76
CF%	3.56	3.36	3.15
Ca%	0.96	0.87	0.81
Available P%	0.48	0.44	0.41
Lysine%	1.28	1.15	1.06
Methionine%	0.51	0.47	0.45
Threonine%	0.86	0.80	0.74

ME: metabolizable energy; CP: crude protein; EE: ether extract; CF: crude fiber; Ca: calcium; P: phosphorus. * Vitamin and mineral premix per kg of diet: vitamin A, 12,000 IU; vitamin D3, 5000 IU; vitamin E, 80 IU; vitamin K3, 3.2 mg; thiamine, 3.2 mg; riboflavin, 8.6 mg; pantothenic acid, 20 mg; folic acid, 2.2 mg; pyridoxine, 4.3 mg; niacin, 65 mg; vitamin B12, 0.017 mg; biotin, 0.22 mg; choline, 1650 mg; Fe, 20 mg; Cu, 16 mg; Mn, 120 mg; Zn, 110 mg; I, 1.25 mg; Se, 0.30 mg.

**Table 2:** Primer sequences and target genes used for real-time qPCR reactions.

Gene	Primer Sequence (5′-3′)	Accession No.
MSTN	F: ATGCAGATCGCGGTTGATC R: GCGTTCTCTGTGGGCTGACT	NM_001001461.1
MyoD	F: CAGCAGCTACTACACGGAATCA R: GGAAATCCTCTCCACAATGCTT	NM_204214.2
Myogenin	F: GGAGAAGCGGAGGCTGAAG R: GCAGAGTGCTGCGTTTCAGA	NM_204184.1
mTOR	F: CATGTCAGGCACTGTGTCTATTCTC R: CTTTCGCCCTTGTTTCTTCACT	XM_417614.5
IGF-1	F: GCTGCCGGCCCAGAA R: ACGAACTGAAGAGCATCAACCA	NM_001004384.2
Pax3	F: ACTACCCTGACATTTATACTCG TGCCTGCTTCCTCCATCTAG	NM_204269.1
Pax7	F: AGGCTGACTTCTCCATCTCTCCT R: TGTAACTGGTGGTGCTGTAGGTG	XM_015296832.1
House keeping		
GAPDH	F: CAACCCCCAATGTCTCTGTT R: TCAGCAGCAGCCTTCACTAC	NM205518
U6	TTCAGGCTCTTGGACGATTT CCGCTATTCCCAAGACTGAA	NM_001277862.1

MSTN: myostatin; MyoD: myogenic determination factor; MyoG: myogenin; mTOR: mechanistic target of rapamycin; IGF-1: insulin-like growth factor 1; Pax: paired-box.; GAPDH: glyceraldehyde-3-phosphate dehydrogenase.

**Table 3:** The primer sequences for microRNA real-time reverse transcription qPCR.

MicroRNA	Primer Name	Primer Sequence
gga-mir-1a	Stem-loop RT	5′-GTTGGCTCTGGTGCAGGGTCCGAGGTATTCGCACCAGAGCCAACTATGGG-3′
gga-mir-1a	Forward	5′-TGGGGGGGACATACTTCTTTATATG-3′
gga-mir-27b	Stem-loop RT	5′-GTTGGCTCTGGTGCAGGGTCCGAGGTATTCGCACCAGAGCCAACTGTTCA-3′
gga-mir-27b	Forward	5′-GGTTTTTTTTAGAGCTTAGCTGATTGG-3′
gga-mir-133a	Stem-loop RT	5′-GTTGGCTCTGGTGCAGGGTCCGAGGTATTCGCACCAGAGCCAACGATTTG-3′
gga-mir-133a	Forward	5′-GGTGTTTTTAGCTGGTAAAATGGAAC-3′
gga-mir-133b	Stem-loop RT	5′-GTTGGCTCTGGTGCAGGGTCCGAGGTATTCGCACCAGAGCCAACTAGCTG-3′
gga-mir-133b	Forward	5′-TTTTTGTTTTTTGGTCCCCTTCAAC-3′
gga-mir-206	Stem-loop RT	5′-GTTGGCTCTGGTGCAGGGTCCGAGGTATTCGCACCAGAGCCAACCCACAC-3′
gga-mir-206	Forward	5′-GTTTGGTGTGGAATGTAAGGAAGT-3′
gga-mir-499	Stem-loop RT	5′-GTTGGCTCTGGTGCAGGGTCCGAGGTATTCGCACCAGAGCCAACCTAAAC-3′
gga-mir-499	Forward	5′-TGGTTTTTTGGTTAAGACTTGTAGTGAT-3′

**Table 4:** Effect of GHBP on the growth performance of broiler chickens.

Parameter	Control	GHBP100	GHBP200	GHBP300	SEM	*p*-Value
Starter (1–10 days)						
BW, g	328 ^b^	335 ^a^	339 ^a^	336 ^a^	5.64	<0.001
BWG, g	283 ^b^	290 ^a^	295 ^a^	292 ^a^	<0.001	0.01
FI, g	361 ^a^	361 ^a^	346 ^c^	353 ^b^	4.38	0.03
FCR	1.28 ^a^	1.23 ^b^	1.17 ^c^	1.21 ^b^	6.98	0.02
Grower (11–22 days)						
BW, g	1216 ^d^	1240 ^c^	1281 ^a^	1269 ^b^	33.46	0.02
BWG, g	888 ^b^	905 ^b^	942 ^a^	933 ^a^	28.47	<0.001
FI, g	1606 ^d^	1570 ^c^	1501 ^a^	1538 ^b^	45.31	0.03
FCR	1.81 ^a^	1.74 ^b^	1.59 ^d^	1.65 ^c^	<0.001	<0.001
Finisher (23–38 days)						
BW, g	2321 ^d^	2456 ^c^	2714 ^a^	2576 ^b^	43.18	<0.001
BWG, g	1105 ^d^	1216 ^c^	1433 ^a^	1307 ^b^	34.44	<0.001
FI, g	2309	2321	2433	2380	39.18	0.30
FCR	2.09 ^a^	1.91 ^b^	1.70 ^c^	1.82 ^b^	0.03	<0.001
Overall performance (1–38 days)						
BW, g	2321 ^d^	2456 ^c^	2714 ^a^	2576 ^b^	43.18	<0.001
BWG, g	2276 ^d^	2411 ^c^	2670 ^a^	2532 ^b^	55.65	<0.001
FI, g	4276	4248	4280	4271	46.18	0.964
FCR	1.88 ^a^	1.76 ^b^	1.60 ^d^	1.69^c^	<0.001	<0.001

^a,b,c,d^ Means with different superscripts within the same row differ significantly (*p* < 0.05). GHBP: growth hormone-boosting peptide; BW: Body weight; BWG: Body weight gain; FI: Feed intake; FCR: Feed conversion ratio. Control: basal diet without GHBP; GHBP100: basal diet plus GHBP at a level of 100 μg/kg; GHBP200: basal diet plus GHBP at a level of 200 μg/kg; GHBP300: basal diet plus GHBP at a level of 300 μg /kg diet.

**Table 5:** Effect of dietary GHBP on serum biochemical parameters of broiler chickens.

Parameter	Control	GHBP100	GHBP200	GHBP300	SEM	*p*-Value
ALT, U/L	37.9	36.8	36.2	39.1	0.520	0.217
AST, U/L	36.6	34.3	33.1	33.2	0.650	0.210
Uric acid, μmol/L	11.2	11.1	13.0	13.5	0.589	0.390
Creatinine, mg/dL	0.63	0.70	0.68	0.71	0.016	0.316
Cholesterol, mg/dL	98.4 ^a^	90.7 ^b^	89.9 ^b^	83.3^c^	1.68	<0.001
TGs, mg/dL	87.6	85.9	89.6	82.9	1.18	0.241
HDL-C, mg/dL	34.9 ^c^	41.1 ^b^	41.7 ^b^	52.0 ^a^	1.93	<0.001
LDL-C, mg/dL	45.9 ^a^	32.4 ^b^	30.4 ^b^	14.7 ^c^	3.43	<0.001
VLDL-C, mg/dL	17.5	17.2	17.9	16.6	0.235	0.241
IGF-1 day 20, ng/mL	9.53 ^c^	15.27 ^b^	18.50 ^a^	13.55 ^b^	1.01	<0.001
IGF-1 day 38, ng/mL	10.73 ^c^	16.47 ^b^	19.70 ^a^	14.75 ^b^	1.00	<0.001
GH day 20, ng/mL	6.87 ^b^	7.67 ^b^	10.07 ^a^	9.52 ^a^	0.438	0.003
GH day 38, ng/mL	8.80 ^c^	10.20 ^b^	12.30 ^a^	10.73 ^b^	0.390	<0.001

^a,b,c^ Means with different superscripts within the same row differ significantly (*p* < 0.05). GHBP: growth hormone-boosting peptide; ALT: alanine aminotransferase; AST: aspartate aminotransferase; TGs: triglycerides; HDL-C: high-density lipoprotein cholesterol; LDL-C: low-density lipoprotein cholesterol; VLDL-C: very-low density lipoprotein cholesterol; IGF-1: insulin-like growth factor 1; GH: growth hormone. Control: basal diet without GHBP; GHBP100: basal diet plus GHBP at a level of 100 μg/kg; GHBP200: basal diet plus GHBP at a level of 200 μg/kg; GHBP300: basal diet plus GHBP at a level of 300 μg /kg.

## Data Availability

The data presented in this study are available on request from the corresponding author.
